# Current Techniques of Water Solubility Improvement for Antioxidant Compounds and Their Correlation with Its Activity: Molecular Pharmaceutics

**DOI:** 10.3390/antiox12020378

**Published:** 2023-02-04

**Authors:** Arif Budiman, Agus Rusdin, Diah Lia Aulifa

**Affiliations:** 1 Department of Pharmaceutics and Pharmaceutical Technology, Faculty of Pharmacy, Universitas Padjadjaran, Bandung 45363, Indonesia; 2 Department of Pharmaceutical Analysis and Medicinal Chemistry, Faculty of Pharmacy, Universitas Padjadjaran, Bandung 45363, Indonesia; 3 Department of Pharmacy, Poltekkes Kemenkes Bandung, Bandung 40161, Indonesia

**Keywords:** antioxidant, poorly water-soluble, water solubility improvement, molecular pharmaceutics

## Abstract

The aqueous solubility of a drug is important in the oral formulation because the drug can be absorbed from intestinal sites after being dissolved in the gastrointestinal fluid, leading to its bioavailability. Almost 80% of active pharmaceutical ingredients are poorly water-soluble, including antioxidant compounds. This makes antioxidant activity inefficient in preventing disease, particularly for orally administered formulations. Although several investigations have been carried out to improve the solubility of antioxidant compounds, there is still limited research fully discussing the subject. Therefore, this study aimed to provide an overview and discussion of the issues related to the methods that have been used to improve the solubility and activity of antioxidant compounds. Articles were found using the keywords “antioxidant” and “water solubility improvement” in the Scopus, PubMed, and Google Scholar databases. The selected articles were published within the last five years to ensure all information was up-to-date with the same objectives. The most popular methods of the strategies employed were solid dispersion, co-amorphous, and nanoparticle drug delivery systems, which were used to enhance the solubility of antioxidant compounds. These investigations produced impressive results, with a detailed discussion of the mechanism of improvement in the solubility and antioxidant activity of the compounds developed. This review shows that the strategies used to increase the solubility of antioxidant compounds successfully improved their antioxidant activity with enhanced free radical scavenging abilities.

## 1. Introduction

Degenerative diseases are the most common type of disease in the world, and they can affect every human being due to the fact of aging. Poor lifestyle habits and the presence of oxidative stress, where the number of free radicals in the body exceeds the number of antioxidants, also contribute to the incidence of degenerative diseases [1].

Free radicals are reactive molecules, also referred to as unpaired electron compounds, which are produced or enter the body due to the presence of a variety of internal and environmental triggers. Proteins, unsaturated weak acids, and lipoproteins, as well as DNA constituents, such as carbohydrates, are free radicals’ primary targets. When free radicals bind with those targets, it will induce abnormal functioning of the cell, including mutation [2].

Antioxidants are chemical compounds that can stop, delay, or completely reverse oxidative reactions by giving the electron to the free radicals, leading to the reduction of the unstable compound. Both natural and synthetic antioxidants are obtained from nature (i.e., plants and animals) or chemically synthesized, respectively. The ability of these compounds has been proven to prevent generative diseases.

However, some physicochemical limitations related to solubility can influence the bioavailability and effectivity of antioxidants [3]. The aqueous solubility of a drug for an oral formulation is very important, because it has a strong influence on the drug’s bioavailability. The drug molecules could be absorbed after dissolving in gastrointestinal fluid [4,5,6]. Therefore, developing a strategy to increase the solubility of a drug is necessary for the formulation of a poorly water-soluble drug, especially for oral antioxidant drug formulations [7,8].

Currently, several physical and chemical modifications have been investigated to improve the water solubility property of antioxidants. These include structure modification, co-crystallization, co-amorphous, inclusion complexes, and nanoparticles, as well as a solid and amorphous solid dispersions (ASDs). However, there is no detailed information on the improvement of the solubility and activity of the antioxidant compound. Therefore, this study aimed to summarize and discuss the solubility improvement techniques that have been investigated for antioxidant compounds. The results are expected to be more valuable due to the objectives and the novelty of explaining the molecular pharmaceutic mechanisms and how the modifications affect the solubility, bioavailability, and effectivity [9,10,11].

## 2. Methodology

**Figure 1:**
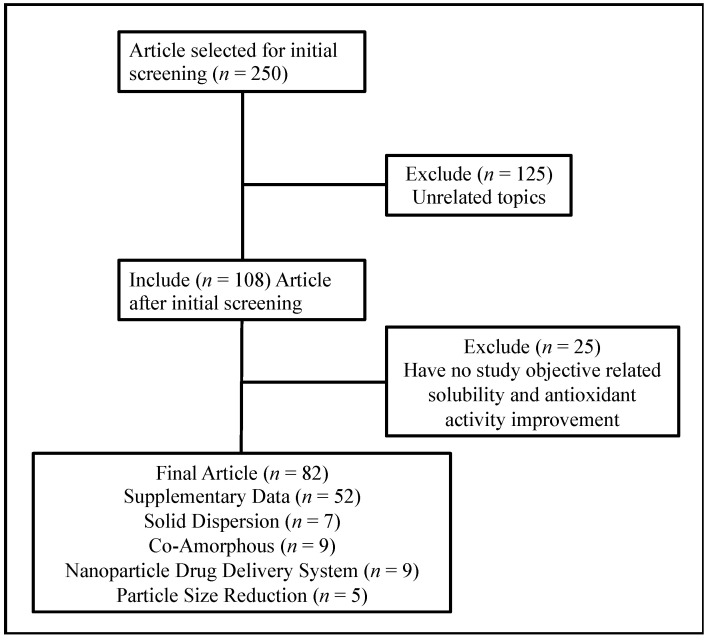
Flow chart of the methodology.

This study was conducted based on the literature obtained from the Scopus, PubMed, and Google Scholar databases since 2018, using the keywords “antioxidant” and “solubility improvement”. Opinions, reviews, and unrelated topics were excluded, and the databases were limited to obtaining the specific topic of pharmaceutical formulations related to water solubility improvement strategies on antioxidant compounds. A flow chart of the methodology is shown in Figure 1.

## 3. Antioxidant

Antioxidants serve as the body’s defensive mechanism against reactive oxygen species (ROS) [12]. Furthermore, they are chemicals that can significantly slow down the oxidation reaction of a substrate in low concentrations. Previous studies asserted that by giving electrons to a radical molecule known to have an unpaired electron, antioxidants can delay, prevent, or completely reverse the oxidative damage to a target molecule. Carotenoids, flavonoids, chalcones, tannins, stilbenes, xanthones, and vitamins are the current categories for the active components that act as antioxidants, as shown in Table 1.

### 3.1. Synthetic Antioxidants

Synthetic antioxidants are synthesized chemically because they are unavailable. Furthermore, they are typically purposefully included in food ingredients as preservatives to stop lipid oxidation reactions [28].

### 3.2. Natural Antioxidants

Natural antioxidants are composed of chemicals found in fruits and vegetables, and they have recently attracted scientific and general interest for study as well as for practical application due to the fact of their properties and benefits [29]. Several types of natural antioxidants gained through daily food intake are well known for their benefits, and they can be regularly ingested with a staple food [32]. The antioxidants that are widely present in food and for which the advantages have also been investigated include vitamin C, vitamin E, beta-carotene, lycopene, and lutein. Vitamin C is water-soluble and primarily functions to neutralize ROS in the aqueous phase to prevent lipid peroxidation in extracellular fluid [33]. It was also reported that vitamin E, a significant fat-soluble antioxidant, has effectively broken down chain reactions in cell membranes, thereby protecting fatty acids in cell membranes from lipid peroxidation. Furthermore, it is assumed that the antioxidant properties of beta-carotene and other carotenoid-derived compounds protect lipid-rich webs. According to previous investigations, beta-carotene and vitamin-class antioxidants act with each other to inhibit lipid peroxidase [34,35].

### 3.3. Antioxidant Function

According to the Food and Drug Administration (FDA), antioxidants are compounds that are only used as dietary supplements and must be consumed in addition to a normal diet to prevent the development or escalation of diseases. According to investigations, eating a diet high in antioxidants, such as fruits and vegetables, can improve long-term health. Recently, antioxidants have gained much attention because of their proven effectiveness in preventing malignancies brought on by oxidative stress due to the fact of exposure to many free radicals [36]. In the body, these compounds collaborate as a system to protect cells from free radicals and the harmful product of their metabolites. There is a potential mechanism by which antioxidants act to slow down the rate of oxidation of fats and oils due to the presence of hydrogen from the antioxidant molecule, which can act as the hydrogen bonding donor for free radicals to make it more stable and inhibit the oxidation of fats caused by free radicals. As they create antioxidants first in the sequence, antioxidants act as electron donors, and after the addition of lipids, complexes are formed between both compounds [37,38]. 

**Figure 2:**
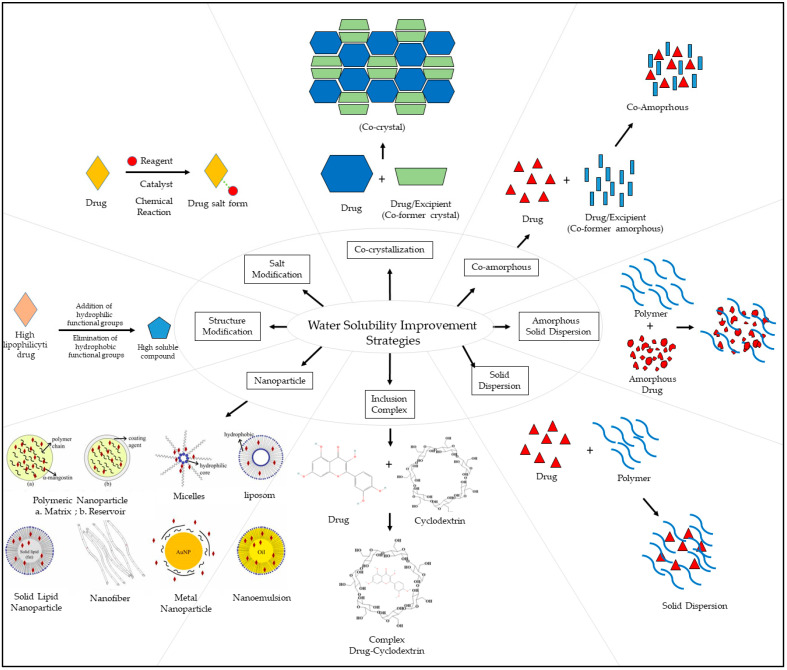
Water solubility improvement strategies overview.

Some antioxidant compounds have restrictions on their physicochemical properties, such as solubility. Aqueous solubility is very important, specifically for orally administered formulations. This is because after dissolving in gastrointestinal fluid, they can be absorbed from the intestine, leading to a strong influence on the bioavailability. However, the poor water solubility of antioxidant compounds is the main hindrance in the development of orally administered formulations due to the lipophilicity of a compound. This causes a low amount of antioxidants in the biorelevant dissolution media, which affects the smallest quantity of active antioxidants and influences their activities. Therefore, various strategies were developed to address the solubility of antioxidant compounds to increase their activity. The general strategies used to improve the solubility of chemicals that are poorly soluble in water are stated below (Figure 2).

## 4. Current Water Solubility Improvement Strategies for Poorly Soluble Drugs

### 4.1. Chemical Modification

#### 4.1.1. Salt Modification

One of the solubility enhancement approaches that employs the principle of chemical structure modification is the synthesis of less water-soluble salts on the active ingredient [38]. The results show that the active ingredient that has been modified into its salt will dissolve significantly faster than the original form. This is because the salt’s ionization in the solvent medium occurs more quickly [39]. The techniques for salt modification are typically used on bioactive substances that are poorly soluble in water. The solubility of the active substance can be increased by salt modification; however, under some conditions, the salt formed is less stable [40]. Although salt modification increases a drug’s solubility in water, the method must be customized to the dissolution medium that is sought.

#### 4.1.2. Structure Modification

**Figure 3:**
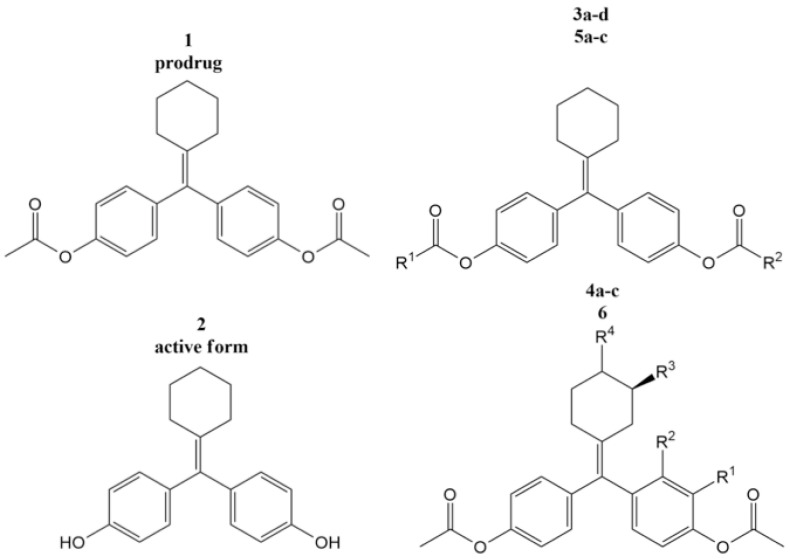
Chemical structures of cyclofenil (**1**), its active form **2**, asymmetric analogs **3a**–**d** and **4a**–**c**, and symmetric analogs **5a**–**c** and **6**. Adapted from data presented originally in Ref. [44].

Structural modification is frequently used in active pharmaceutical ingredient (API) development to improve solubility, dissolution, and bioavailability [41]. The generation of a new drug is the ultimate goal of structural modification; therefore, during development and invention, pharmacological activity and drug stability are two key considerations [42]. For pharmacokinetics and drug safety, the physicochemical and biochemical parameters are modified to enhance the pharmacological activity and stability. The fundamental concept behind chemical structure modification to increase solubility is to focus on adding or replacing hydrophilic groups, namely, carboxylate, amine, ester, ketone, and hydroxy, in the structure of active pharmaceutical compounds when creating new molecules [43]. Figure 3 shows that the introduction of a chiral methyl group in (*R*)-**4c** on the structure of cyclofenil can improve its solubility by approximately 3.6 times compared to structure 1. This is due to the introduction of the chiral center, which can lead to greater molecular asymmetry [44].

### 4.2. Physical Modification

#### 4.2.1. Particle Size Reduction

**Figure 4:**
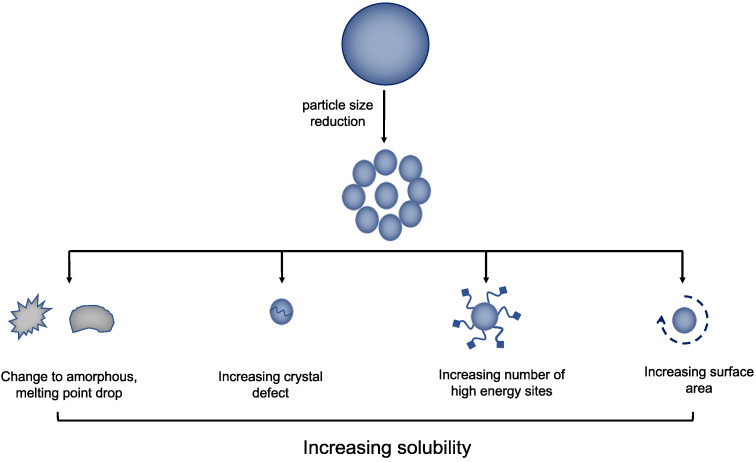
The relationship between particle size reduction of drug and its solubility enhancement. Adapted from data presented originally in Ref. [49].

Particle size reduction is a common approach used in medication manufacturing or API development in order to address the psychochemical properties issue of poorly water-soluble drugs [45]. However, the disadvantages are the prolonged manufacturing time and the requirement for micronization at an early stage of the production process. Particle size reduction is often separated into two categories, namely, bottom-up and top-down. Bottom-up refers to a process where a substance is first changed into a molecular state using an appropriate solvent and slowly recrystallized by transforming from a molecular state into particulate crystals [46]. Meanwhile, the top-down technique, which is more common, entails lowering the particle size through the milling process or other appropriate operations. To obtain the best results during particle size reduction, top-down and bottom-up approaches are frequently combined [47]. This method aims to improve the solubility by decreasing the particle size and increasing the surface area. Since large surfaces allow for greater contact between the particles and the solvent medium, they will become more wettable quickly and dissolve the active ingredient faster [48]. Alshora et al. (2016) reported that the increased solubility of a poorly water-soluble drug by particle size is caused by several factors, as shown in Figure 4. Firstly, reducing the particle size can raise the surface-to-volume ratio, leading to an increase in the particle surface exposed to the solvent and improving the solubilizing activity by increasing the solvent contact area. Secondly, the small size can increase the number of high-energy sites, which creates more opportunities for the solid particle to interact with the solvent. Thirdly, the method also produces crystal defects, leading to the easy removal of molecules from weak particles. Finally, the smaller particle size reduces its melting point, which results in fewer intramolecular interactions of the drugs and a higher solubility [49].

#### 4.2.2. Nanoparticle Drug Delivery System

Nanomedicine is the application of nanotechnology that plays a major role in clinical therapy. Due to the fact of their nanosize (1–100 nm), nanocompounds have a large surface area, which increases the surface contact with the solvent and increases the solubility or rate of dissolution of less water-soluble compounds [50]. Therapeutic nanomedicine interventions can be extremely precise at the intermolecular scale, enabling the treatment of diseases or the restoration of harmed tissues, such as bones, muscles, or nerves. Examples of nanocarriers that have been developed as drug delivery systems include liposomes, dendrimers, solid lipid nanoparticles, polymeric nanoparticles, silicon or carbon materials, metal, and magnetic nanoparticles [51]. A possible modification method is a drug delivery system based on nanoparticles, which combines physics and chemical disciplines. It is a tried-and-true advantageous method to overcome the problem of pharmaceuticals with low water solubility and provides a targeted drug delivery system [52].

#### 4.2.3. Co-Crystallization

**Figure 5:**
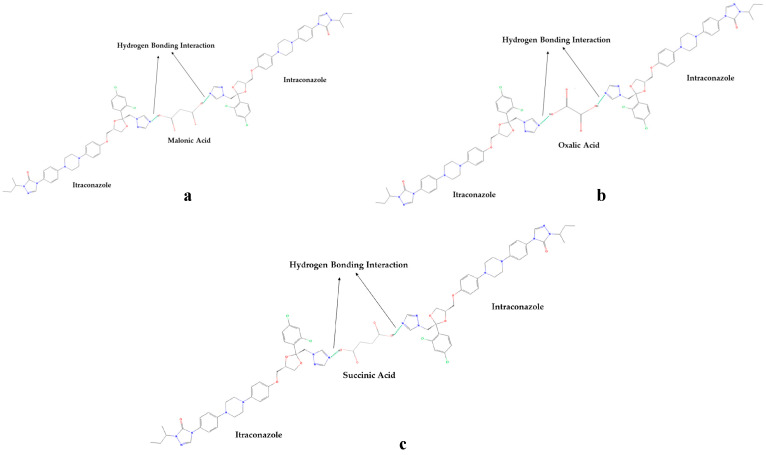
Hypothetic trimers can be formed from co-crystal of ITZ and dicarboxylic acids with various carbon chain lengths, (**a**) ITZ-Malonic Acid, (**b**) ITZ-Oxalic Acid, and (**c**) ITZ-SCU. Adapted from data presented originally in Ref. [59].

A co-crystal is a multicomponent crystal that contains noncovalent interactions, such as hydrogen, van der Waals, and ionic bonds, in a crystal lattice, where all of the components are typically solid at room temperature in a stoichiometric ratio [53]. Along with the API, co-crystals include pharmaceutically approved guest molecules in a crystal lattice. Pharmaceuticals’ physiochemical properties can be enhanced by co-crystallization to produce co-crystals. The pharmacological activity of the API was not affected by co-crystallization with pharmaceutically acceptable (GRAS) chemicals, but it can improve the physical properties, including the solubility, hygroscopicity, and compaction behavior [54]. Pharmaceutical co-crystals are crystalline substances that include an API and one or more distinctive co-crystal formers and are solids at room temperature. Crystal engineering can be used to enhance physical and chemical properties, which is beneficial for pharmaceutical co-crystals. Previous investigations showed that an API and a co-crystal former are two neutral compounds that are combined into a single crystalline solid to generate a pharmaceutical co-crystal [55]. Co-crystals are created through a variety of interactions, such as hydrogen bonds, π–π stacking, and van der Waals forces. Although API solvates and hydrates are not considered co-crystals under this definition, the crystal lattice of co-crystals may contain one or more molecules of solvent or water. Co-crystals improve pharmaceutical properties by altering the solubility, dissolving rate, mechanical behavior, moisture uptake, physical and chemical stability, and bioavailability of nonionizable drugs without changing their pharmacological action [56,57]. The review topics included the growing range of crystal form options, the rise of crystal engineering in the field of pharmaceutical science, and pharmaceutical co-crystals. Furthermore, the aspects of the co-crystal formation that were frequently encountered, the screening tactics, and the conceptual methodologies for the co-crystal functionality were also reported. Recently, there has been much interest in the use of co-crystals in drug delivery and design as well as functional materials with prospective applications in medicines [58]. Figure 5 shows the mechanism of the co-crystal formation between itraconazole (ITZ) and succinic acid (SUC). The crystal structure of ITZ-SUC is dominated by supramolecular trimers, consisting of two molecules with a head-to-tail orientation and a SUC molecule, as presented in Figure 5a. The molecule of SUC is located in the pocket that is formed by two ITZ molecules and H bonded with the 1,2,4-triazole groups serving as a bridge between the two molecules (host). The SUC was replaced with the dicarboxylic acids, and the ITZ molecules were added to the acid; therefore, the H bonding and ITZ geometry molecule stayed the same as in the ITZ-SUC co-crystal. The conformations of the dicarboxylic acids (Figure 5b–cwere selected to ensure the pocket geometry of ITZ-SUC in the model structures.

#### 4.2.4. Co-Amorphous

**Figure 6:**
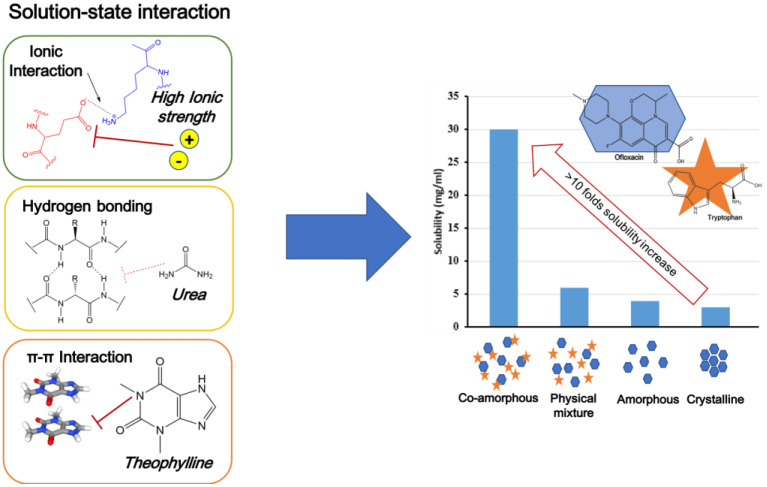
The formation of a co-amorphous system between ofloxacin and tryptophan. Adapted from data presented originally in Ref. [65].

Co-amorphous is the stabilization of a drug’s amorphous form by one or more low-molecular-weight excipients and/or other drug molecules, the co-former(s), and their formation of a homogenous single-phase amorphous system [60]. Compared to polymer-based and mesoporous silica-based, ASD has certain advantages because the drug load is often raised from a 20 to 30 weight percent to 50 or higher. The low-molecular-weight co-formers can be additional pharmaceutical ingredients or low-molecular-weight excipients, such as amino acids, organic acids, and other tiny molecules, such as nicotinamide. The co-former physically stabilizes the drug’s amorphous form or interacts with it on a molecular level by salt formation, hydrogen bonding, and π–π interactions [61,62]. Co-amorphous drug delivery systems (CAMS) can be defined by three critical quality attributes (CQAs), namely, co-formability, physical stability, and dissolution performance. The first CQA discusses the potential for creating a CAMS using a medication and a predetermined co-former in a ratio for a known preparation process [63]. Physical stability is described as the second CQA, since the CAMS must increase it for the pure drug’s amorphous, thermodynamically unstable state [64]. Finally, the third CQA must be evaluated, which is the degree of dissolution enhancement in comparison to the pure drug in the crystalline and amorphous forms. A better bioavailability in vivo can be obtained from achieving and maintaining supersaturation, which is the definition of enhanced dissolving performance. Figure 6 shows the co-amorphous system of ofloxacin with tryptophan. The amorphization of co-amorphous was confirmed as a halo pattern and a single glass transition event in the powder X-ray diffraction measurements and the modulated differential scanning calorimetry (MDSC) curve, respectively. The hydrogen bonding and π–π stacking in the solid state of the co-amorphous system was observed by Fourier transform infrared spectroscopy measurement.

#### 4.2.5. Amorphous Solid Dispersion (ASD)

**Figure 7:**
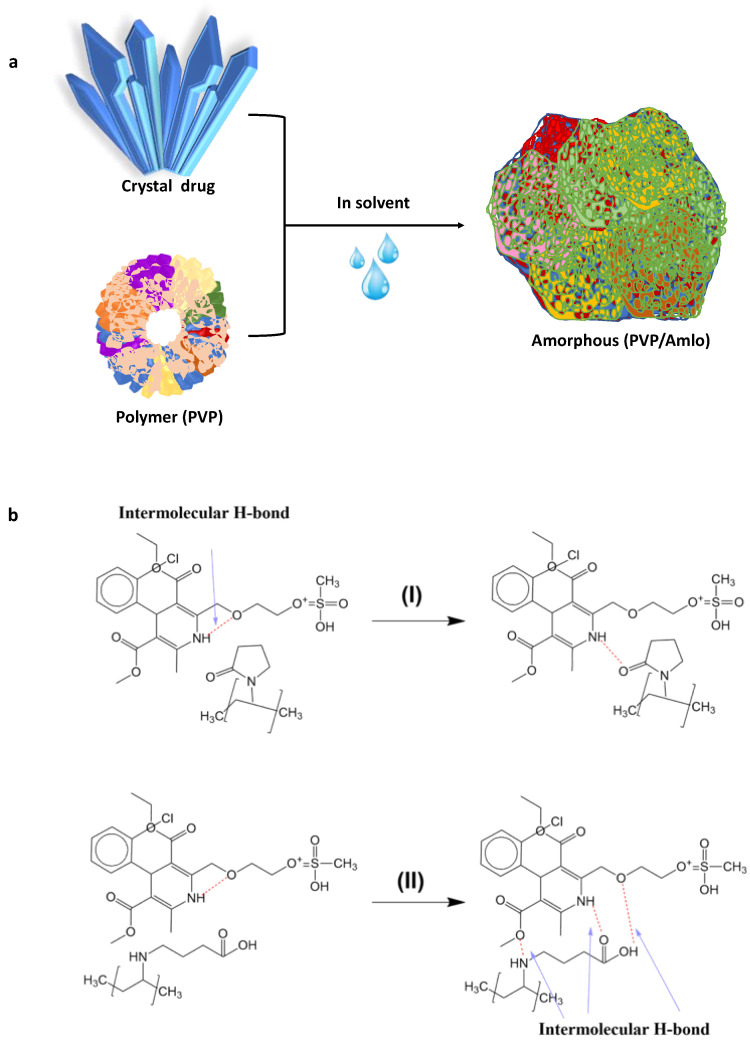
The schematic illustration of (**a**) hydrophilic and hydrophobic sides of the amlodipine besylate. (**b**) System of amorphous solid dispersion. Formation mechanism of drug–drug (I) and drug–polymer (II) intramolecular and intermolecular hydrogen bonding, respectively. H bonds are shown as red lines. Adapted from data presented originally in Ref. [70].

A promising formulation strategy to increase the solubility, rate of dissolution, and bioavailability of pharmaceuticals that dissolve slowly in water is called ASD. Due to the varied production methods and formulations, ASD has complex physicochemical properties [66]. Since these characteristics affect their physical stability, it is necessary to create efficient characterization methods for ASD. ASD has also been used in modern formulation development to increase the bioavailability of less water-soluble medications. However, the physical instability of ASD limits the possibility of their use in commercial products [67]. Drug solubility is determined by the chemical potential, and in ASD systems, the free energy is raised due to the numerous energy inputs, which causes the crystalline form to change into an amorphous state. Comparing ASD to its crystalline form, which has lower molecular energy and stronger molecular bonds (mainly ionic) and is more difficult-to-break, ASD is thermodynamically unstable [68]. As a matrix-forming excipient for ASD, the polymer can prevent amorphous pharmaceuticals from recrystallizing by demonstrating increased viscosity when the temperature is below the glass transition temperature (*T_g_*) or by interfering with the interactions between the polymer and the drug [69]. ASDs return to their crystalline state, albeit slowly; ideally, they will be kinetically stable in their predicted shelf life. The presence of water also increases their mobility when exposed to moisture, for example, during storage under high humidity conditions, and decreases the capacity of polymers to inhibit recrystallization. Figure 7a shows the dissolution process for polymer–drug molecules. It was discovered that the structure of the drug formed was crystalline, where the drug–drug interactions were strong enough due to the intramolecular H bonds and could lower the solubility. Figure 7b reveals the formation of the polymer–drug intermolecular H bond interaction, showing (I) the interaction between drug–drug interaction, which is intermolecular H bonds with polymers caused by the stability of the solid dispersion. (II) The drug–polymer intermolecular hydrogen bonding uses three hydrogen donor groups of polymer molecules [70].

#### 4.2.6. Solid Dispersion

**Figure 8:**
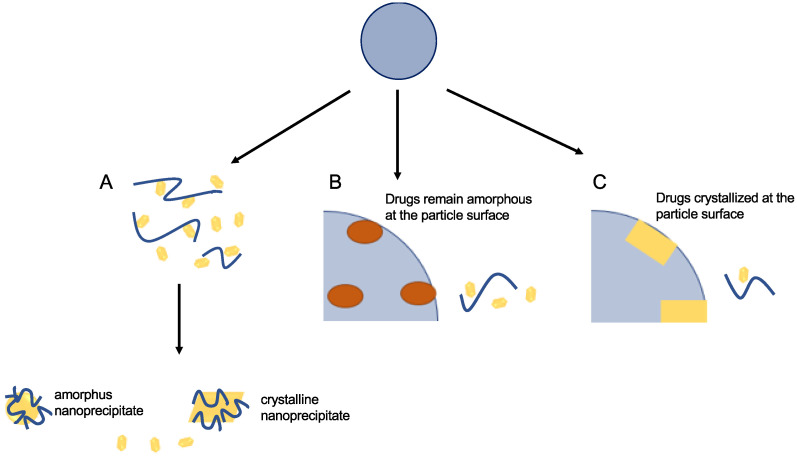
Three possible scenarios of drug dispersion from solid dispersions. (**A**) Particles rapidly dissolve and release the drug into a highly supersaturated solution; (**B**) drug and polymer are gradually released; and (**C**) drug and polymer are gradually released but the drug is present in a crystals state. Adapted from data presented originally in Ref. [74].

A solid dispersion is “a dispersion involving the production of eutectic mixes of pharmaceuticals with water-soluble carriers by melting of their physical mixtures”, according to Chiou and Riegelman [68]. The dispersion of one or more active ingredients in an inert carrier or matrix at a solid state created by melting (fusion), solvent, or the melting solvent method is referred to as solid dispersion. Sekiguchi et al. also stated that after a few years, the drug can be presented in a microcrystalline condition as a eutectic combination. According to Goldberg et al., not all drugs in solid dispersion are necessarily present in a microcrystalline state. This is because certain drugs can be molecularly dispersed in the matrix to form solid solutions [71,72]. The medication is released as exceptionally small, colloidal particles after the solid dispersion is exposed to watery solutions and the carrier disintegrates. Therefore, poorly water-soluble drugs are anticipated to dissolve quickly and have a high bioavailability due to the significantly increased surface area obtained. Surface active and self-emulsifying carriers can also be used to solve manufacturing issues with solid dispersion systems, which have largely limited the commercial usage of such systems [73]. The medications are dissolved in melted carriers when heated to a high temperature. A collection of solid products with at least two separate components, often a hydrophilic matrix and a hydrophobic drug, are referred to as solid dispersion, where the matrix is crystalline or amorphous. The drug can be spread molecularly, in crystalline or amorphous particles (clusters). Figure 8 shows the type of solid dispersion that can occur in a dissolution medium. Type I in Figure 8A indicates that the drug and polymer in the solid dispersion rapidly dissolved in the dissolution medium due to the low drug loading. Therefore, the drug is continuously absorbed and passes through the precipitation in the presence of the polymer and endogenous compounds, such as phospholipids, bile acids, and mucin. Solid dispersion particles can slowly dissolve due to the high drug loading or the nature of the polymer, leading to a more sustained release profile. When the water penetrates the particles of a solid dispersion, phase separation will occur. As shown in Figure 8B, when the drug crystallization is still inhibited by the polymer matrix, the drug must be in amorphous aggregates, and the free drug concentration will be equal to the solubility of the amorphous drugs in the dissolution media. However, when the drug is in a crystalline state in the solid dispersion, the free drug concentration in the dissolution medium decreases to that of the solubility of the drug crystals, as presented in Figure 8C.

#### 4.2.7. Toxicology Study of Water Solubility Improvement Strategy

Overall, water solubility improvement strategies have various benefits in addition to their primary objective of increasing solubility and dissolution rate, such as physicochemical properties. One of the approaches, the nanoparticle drug delivery system, is the one that has been found to have the most negative side effects.

Nanoparticles are delivery systems with impressive capabilities in increasing physicochemical properties to improve the effectiveness of a therapeutic substance, beginning with solubility and stability and progressing to the capacity to provide a modified and targeted delivery system. Despite the efficacy of the nanoparticle delivery system, it was discovered that this modification technique also had unfavorable side effects. Numerous studies on the negative consequences of nanoparticle use on several organs, including the heart, liver, and lung, have been published. Regarding the carcinogenic risks of nanoparticles, various additions have also been published. This happens as a result of the buildup of nanoparticles over a prolonged period of time.

1. Liver side effects of nanoparticles

Zhang N. Y et al. conducted a previous study on the influence of nanoparticles on hepatic cells, and the results showed that 30–99% of administered nanoparticles concentrate and sequester in the liver following injection into the body. Due to the decreased delivery to the targeted sick tissue, the hepatic cells may become more toxic as a result. One of the main obstacles to the practical use of nanoparticles is the liver. Nanoparticles cannot target sick tissue outside of the liver due to the sequestration of these particles by the liver. The 1970s and 1980s saw a large number of studies on the subject of particle-to-liver interactions; nevertheless, the results reached at that time may not be entirely applicable to the present nanoparticle technology. Particles that are less than 100 nm in size are comparable in size to many biological compounds. They might have access to various cellular and tissue structures in the liver and might have various patterns of accumulation. On the Kupffer Cells, the surface charge, ligand chemistry, and size of the nanoparticle all have a significant impact on the rate of uptake and retention in the cells. In vitro, nanoparticles with strongly cationic and anionic surface charges exhibit increased macrophage interaction and absorb a large quantity of serum proteins to form a “protein corona” [75].

2. Lung side effects of nanoparticles

The pulmonary effects of single-walled carbon nanotubes were demonstrated in vivo after intratracheal instillation in both rats and mice, according to a recent paper on the toxicity of nanotubes. The results showed that carbon nanotubes are substantially more hazardous than carbon black and may even be more harmful than quartz if they enter the lungs. Both groups experienced granuloma development and some interstitial inflammation. Rats were subchronically exposed for three months to ultrafine (20 nm) and fine (200 nm) titanium dioxide (TiO_2_) particles, and it was shown that the ultrafine particles cleared substantially more slowly and showed greater translocation to interstitial locations and local lymph nodes. It is interesting that the low exposure (10 mg/m^3^) research led to a higher incidence of lung tumors than the high exposure (250 mg/m^3^) trial when comparing the health effects of chronically inhaling TiO_2_ particles of clearly different diameters [76,77,78,79].

3. Skin side effects of nanoparticles

According to some reports, human stratum corneum can be penetrated by TiO_2_ particles smaller than a micrometer, and in some cases, these particles can even reach the deeper regions of hair follicles. After being applied to human skin, emzaloid particles—a submicron emulsion particle having a diameter of 50 nm to 1 micron and resembling liposomes and nonionic surfactant vesicles (niosomes)—were found in the epidermis along with the cell membranes. The authors hypothesized that the single molecules that make up the particles may enter the crevices between cells and, in specific places in the stratum corneum, could build up and reform into tiny spheres [80,81].

4. Body Distribution and Systemic Side effects of nanoparticles.

Particle surface properties have a significant impact on how they are distributed throughout bodies. For instance, the distribution of poly (methyl methacrylate) nanoparticles throughout the body is considerably altered by covering them with various types and quantities of surfactants. Thirty minutes after intravenous injection, coating these nanoparticles with 0.1% poloxamine 908 dramatically lowers their liver levels (from 75 to 13% of the total amount of particles administered). Polysorbate 80, another surfactant, proved effective above 0.5%.

5. Nanoparticle and thrombosis

There is a strong correlation between particle air pollution and harmful cardiovascular outcomes including myocardial infarction, according to epidemiological studies. They focused on thrombus development as a pertinent endpoint as they investigated the potential impacts of particles on hemostasis. By intravenously injecting polystyrene particles with a surface charge of neutral, negative, or positive, they have a direct impact on hemostasis. Due to platelet activation, positively charged amine-particles significantly increased prothrombotic tendency. After the intratracheal delivery of these positively charged polystyrene particles, which similarly resulted in lung inflammation, a comparable effect could be attained [82,83].

6. Central nervous system side effect of nanoparticles

Neuron and nanoparticle cultures have been used as in vitro systems to investigate the effects of particles on the nervous system and how they affect neuronal processes. Metal nanoparticles such as Ag, Cu, and Mn have been used in studies on P12 brain cells to look at possible neurotoxicity. Since small particles are more mobile, it is anticipated that either passive diffusion or carrier-mediated endocytosis will be able to move nanoparticles over the BBB. Additionally, trans-synaptic transfer of nanoparticles into the brain is a possibility. For instance, Ag nanoparticles can pass across the BBB and gather in various parts of the brain, which may be advantageous for drug delivery but could potentially be dangerous for the patient [84,85,86,87,88].

7. Membrane cell side effect of nanoparticles

Nanoparticles can have an impact on membrane stability directly (for example, by physical damage) or indirectly (for example, through oxidation), which might result in cell death. The fact that membranes can regulate intracellular homeostasis through mechanisms of selective permeability and transport makes them a prime target for potential negative impacts of nanoparticles. The surface characteristics of the nanoparticles play a significant role in how they interact with membranes. In order to improve the absorption of nanoparticles into cells, surface changes are essential for designing drug delivery systems. The size of the nanoparticles, which affects surface tension and adhesion forces, is also crucial [89,90,91].

8. Nanoparticle effects on mitochondria and lysosomes

Fullerenes and carbon nanotubes appear to be a primary target for mitochondria, which serve as the cell’s power plants. Other nanoparticles, including titanium dioxide, carbon nanotubes, polystyrene, and silver, however, also appear to have the ability to influence mitochondrial function and trigger apoptosis. Lysosomes, the cell’s digestion system, are another preferred intracellular compartment. According to research, nanoparticles frequently find their way into lysosomes, where the cell tries to either digest or expel them. It is unclear how size, shape, and nanomaterial affect a cell’s capacity to ingest or eliminate nanoparticles once they have accumulated in lysosomes [92,93].

9. Nanoparticle effects on proteins and macromolecules

Nanoparticles, which can be the same size as protein molecules, can alter the shape of proteins, such as peptides, causing them to aggregate and fibrillate, or they can interact with proteins by acting as chaperones, which can interfere with cellular signaling pathways. Neuro-degenerative illnesses are characterized by protein misfolding and peptide fibrillation that can produce, for instance, amyloid-like formations. For purposes of understanding nanotoxicological effects, it is crucial to look into potential errors in protein and macromolecule synthesis and excessive cellular production [94,95].

10. Nanoparticle effects on DNA

When evaluating potential toxicological concerns brought on by nanoparticles, DNA has received significant attention. Because scientists have discovered that nanoparticles can infiltrate the nuclear envelope, interest in potential genotoxic effects of nanoparticles has increased. The genotoxicity of several nanoparticles has been investigated. However, it is unclear from these investigations which nanoparticle parameter—positive or negative—is in charge of the results. Additionally, it is unclear how potential DNA damage works. Again, ROS is thought to have a significant role in DNA damage in addition to direct intercalation or the physical and/or electrochemical contact with nanoparticles. This indicates that particles may cause genotoxicity, for instance, by oxidative stress, without necessarily having to enter the nucleus [96,97].

## 5. Water Solubility Improvement Strategies of Poorly Soluble Antioxidants

See Table 2.

### 5.1. Solid Dispersion of Antioxidant Compound

Sa et al. (2018) stated that a curcumin solid dispersion has poor water solubility and low bioavailability, which reduces its usefulness. Their investigation assessed the cytotoxic effects of curcumin solid dispersions (CurSDs) against tumors, such as breast adenocarcinoma, lung, cervical, and hepatocellular carcinoma, as well as nontumor (PLP2) cells. They also determined the influence of a CurSD on the enzymes acetylcholinesterase (AChE), butyrylcholinesterase (BChE), glutathione S-transferase (GST), and monoamine oxidase (MAO A-B). Furthermore, the effectiveness of a CurSD to prevent the development of reactive thiobarbituric acid and oxidative hemolysis (OxHLIA) (TBARS) was examined. The results showed that the nanoparticles that comprised the CurSD easily dissolved in water and also inhibited 24% and 64% of the AChE and BChE activity at 100 M, respectively. The MAO-A and -B activity was suppressed at 100 M, but the GST activity was inhibited at 30 M. Without any harmful effects on the normal cell types, the CurSD demonstrated cytotoxicity against all of the evaluated tumor cell lines. The in vitro manipulation of significant enzymes preserved the properties of the free curcumin without noticeably increasing the toxicity [122].

Moreover, Fitriani et al. (2018) developed a usnic acid solid dispersion with antioxidant properties. The application of this compound is still limited due to the fact of its poor solubility in water. In this investigation, the primary polymer was poly-vinyl-pyrrolidone (PVP) K30, which was produced using the spray and freeze-drying methods with usnic acid:PVP ratios of 1:1 and 1:2 (*w*/*w*). For comparison, a physical mixture with the same proportions was created. The maximum solubility was found in a 1:2 ratio of freeze-dried usnic acid-PVP, which was roughly 20 times more soluble than intact usnic acid. As a result of the antioxidant activity test, the gallic acid, intact usnic acid, and freeze-dried usnic acid showed that all of the parameters at a ratio of 1:2 had IC50 values of 12.471, 80.242, and 63.867 g/mL, respectively. This indicates that usnic acid can become more soluble in solid dispersions, and the antioxidant activity matches the solubility outcome [117].

Crucitti et al. (2018) aimed to create and characterize chitosan and abietic acid (AB) solid dispersions (CS). Based on the results, it was discovered that the dispersion/solvent evaporation technique produced solid dispersions, where the carrier’s abietic acid was molecularly distributed. Particularly in the formulations derived with a 1/1 AB/CS molar ratio, a synergistic impact between the two components in terms of the antioxidant and antibacterial activities were discovered. Furthermore, the antibacterial activity of the formulation was influenced by the aggregation state (amorphous/crystalline) of AB, indicating greater bioactivity when the drug was in the amorphous state. These results appear to open potential vistas for the successful use of the produced AB/CS formulations in the biomedical area or the food industry, with the formulations as shown by biocompatibility [123].

The electrospinning technology and subsequent annealing treatment were used by Salevic et al. (2019) to create new active films consisting of poly(-caprolactone) (PCL) that included a solid sage extract dispersion (SE). The antioxidant and antibacterial properties of the SE were initially verified. This was followed by the assessment of the impact of the SE inclusion on the physicochemical and functional characteristics of the films at loading amounts of 5%, 10%, and 20%. The incorporation of the SE into the PCL matrix produced a significant 2,2-diphenyl-1-picrylhydrazyl (DPPH) free radical scavenging capacity and a robust activity against the foodborne pathogens *Staphylococcus aureus* and *Escherichia coli*. The results indicate that the developed PCL-based films containing SE have several potential uses in active food packaging applications, where they prevent oxidation reactions and microbiological development [124].

Based on the study by Alshehri et al. (2020) on the solid dispersion of luteolin (LT), it was discovered that poorly soluble bioactive chemicals have issues with bioavailability when administered orally. Therefore, enhancing its bioactivity and dissolution became the main objectives of the study. Polyethylene glycol 4000 (PEG 4000) was used to make LT-SD in the mass ratios of 1:1, 1:2, and 1:4. The LT-SD was made using several techniques, including solvent evaporation, fusion, and microwave irradiation. The results reveal that, compared to the pure LT and its physical mixture dispersion, the percentage of LT released from the manufactured SD was much greater after 90 min of the dissolving study (PMD). At a 1:2 mass ratio of LT:PEG 4000, the LT-SD made using the MI technique demonstrated the greatest release of LT (97.78 4.41%). At a mass ratio of 1:4 of LT:PEG 4000, the LT-SD made using the SE technique showed the highest release of 93.78 3.98%. Meanwhile, the SD created using the MI approach showed improved dissolving due to the high water solubility and decreased particle size. An analysis of the antioxidant activity indicated that the LT-SD was significantly more effective in scavenging free radicals than the pure LT. Therefore, increasing the solubility, the in vitro dissolution, and the therapeutic effectiveness of LT can be accomplished with an SD produced using PEG 4000 [10].

Jiang et al. (2020) developed a solid dispersion of *Angelica gigas* Nakai (AGN) using hot-melt extrusion and ultrafine grinding methods. The AGN’s putative anti-inflammatory and antioxidant properties were evaluated using several processes, and the impact on the human Kv1.3 potassium channel was discovered. The total phenolic and flavonoid contents, antioxidant activity, and DNA damage protective impact of the AGN were boosted by the ultrafine powderization method. The human Kv1.3 channel and AGN solid dispersion (AGN-SD) based on Soluplus^®^ had the strongest inhibitory impact on NO generation. The prostaglandin E2 and intracellular reactive oxygen species generation as well as the mRNA expression of the inducible nitric oxide synthase, cyclooxygenase-2, and interleukin 1 and 6 were also suppressed by the AGN-SD. These results showed that AGN’s biological activities can be significantly enhanced by ultrafine powderization and solid dispersion production by hot-melt extrusion (HME). Furthermore, 1e discoveries indicated that HME and ultrafine powderization can be developed and used in the pharma industry [125].

Ma et al. (2020) developed a unique and straightforward procedure for the extraction and identification of seven chemicals from *Anemarrhena asphodeloides* Bge. This was carried out using vortex-homogenized matrix solid-phase dispersion based on silica gel and quadrupole-time of flight mass spectrometry. The extract prepared with the modified procedure was put into a polyamide chromatography column and a D-101 macroporous resin column to screen for possible antioxidants. With the greatest flavonoid content, Fr.2.2 demonstrated the highest antioxidant activity. The active portion yielded 25 peaks for identification. For a quick and accurate screening as well as the identification of antioxidant chemicals, a mass spectrometry technique using 2,2′-diphenyl-1-picrylhydrazyl ultra-high-performance liquid chromatography was used. The results suggest that flavonoids have antioxidant properties. The in vivo tests were also carried out on the antioxidant capacities of nine monomeric molecules, while the relationships between structure and activity were examined. It was discovered that five flavonoids at a dosage of 500 gmL-1 could reduce the oxidative stress that 2,2′-azobis[2-methylpropionamidine] dihydrochloride caused in PC12 cells [98].

Ramadhan et al. (2021) examined a solid dispersion of pentagamavunon-0 (PGV-0), a curcumin analog with antioxidant capabilities. It was synthesized using development procedures employing vanillins of various grades. The PGV-0 has poor bioavailability, but by designing a solid dispersion system, its solubility can be improved. This investigation was carried out to determine the antioxidant capabilities of PGV-0, which was produced using several vanillin-starting materials of varying grades, as well as the impact of PEG 6000 and maltodextrin solid dispersion systems. The results revealed that the PGV-0 of the vanillin pro-analysis group had a stronger antioxidant capacity than the vanillin group (EC50 = 15.35 ppm and EC50 = 13.62 ppm, respectively). Compared to PGV-0-PEG 6000 at a ratio of 1:5 group, the solid dispersion system of PGV-0-PEG 6000 at a ratio of 1:10 showed a robust antioxidant capability (EC50 = 9.00 ppm). Similarly, when compared to PGV-0-maltodextrin at a ratio of 1:5 (EC50 = 13.63 ppm), PGV-0-maltodextrin at a ratio of 1:10 (EC50 = 11.96 ppm) exhibited strong antioxidant activity. This suggests that the antioxidant activity of PGV-0 can be affected by the use of vanillin with various qualities in the compound’s manufacture and solid dispersion systems [99].

### 5.2. Co-Amorphous or Amorphous System

Guo et al. (2018) used a coprecipitation approach to synthesize amorphous calcium phosphate (ACP) nanoparticles to improve the release profile of curcumin (Cur) and minimize burst releases. This was carried out to overcome Cur’s weaknesses, such as low chemical stability and bioavailability. The results demonstrate that amorphous calcium phosphate (ACP) was produced at a pH of 8 and a PO_4_ 3 concentration of 0.024 mM when processed at 30 °C for 10 min. The ACP nanoparticles demonstrated a high loading capacity for Cur and advantageous pH-responsive drug release capabilities in an in vitro drug release experiment. Furthermore, the Cur-loaded ACP nanoparticles demonstrated an outstanding free radical scavenging performance and significant cell viability reduction in A549 cells. This shows that the potential application of nanoparticles in the biomedical and food industries is significant [104].

Himed et al. (2019) also extracted citrus lemon essential oil and analyzed the chemical compounds, antioxidant activity, and the influence of encapsulation on its activity. Based on the results, 1.24 ± 0.07% of the essential oil was obtained from the cold-pressed lemon peel. The composition of this oil was dominated by limonene (67.1%), followed by -pinene (11.0%) and -terpinene (8.0%). The equivalent results were produced by the oil’s antioxidant activity, as measured by the reducing power CUPRAC and the antiradical power DPPH. This oil’s antioxidant activity was unaltered by being enclosed in silica. The encapsulated oil had less thermal stability than the loose oil, according to the thermogravimetric study. Meanwhile, according to FTIR-ATR, the encapsulation did not change the oil’s composition [106].

Guleria et al. (2020) attempted to use a very simple and quick method to resolve such problems in light of the precise synthesis approaches and quick phase change of amorphous-selenium (-Se) nanoparticles. The synthesis approach employed an imidazolium-based room temperature ionic liquid (RTIL), which served as a solvent, as well as a reducing and stabilizing agent. The Se nanoparticles formed within 10 min of the reaction, which was carried out under entirely ambient circumstances. By contrasting these results with those of typical antioxidants, excellent activities of the antioxidant compounds were discovered [107].

A new multifunctional amorphous hydrogel comprising *Olea europaea* leaf extract (OELE) was explored for its antioxidant and wound-healing activities by Diaz et al., in 2022. According to the results of the DPPH, ABTS, and FRAP tests, OELE had significant antioxidant activity with values of 2220, 1558, and 1969 mol TE/g, respectively. Furthermore, an OELE co-treatment enhanced the viability of the HDFs and HaCaT when oxidative damage caused by H_2_O_2_ was corrected, demonstrating a protective function. Compared to the controls, EHO-85 had an early and long-lasting impact that stimulated wound healing in diabetic mice. Due to the high phenolic content of OELE, which shields skin cells from oxidative stress and aids in the physiological process of wound healing, this unique amorphous hydrogel exhibits an essential ROS scavenger capacity [105].

### 5.3. Nanoparticle Drug Delivery System

Merouane et al. (2018) examined the impact of micro- and nanosized particles on the antioxidant activities and the phenolic and flavonoid contents of leaves extracted in aqueous and hydromethane. The results demonstrated that the characteristics of the extracts were unaffected by the removal of microparticles, but the nanoparticles significantly increased the number of total phenolics and the antioxidant potencies of both extracts. The methanolic extract showed the greatest impact of the nanoparticle removal on the antioxidant activity, losing 72% of its ability to block carotene bleaching and quadrupling its half-maximal inhibitory concentration (IC50) index. This shows that nanosized particles are nature’s repository for bioactive components in herbal extracts. Therefore, their maintenance is strongly recommended to retain the full range of antioxidant capabilities [113].

Barky et al. (2020) reported that ellagic acid is a naturally occurring polyphenol that includes potent antioxidants, although it is consumed as a meal that is not well absorbed. To increase its bioactivity and bioavailability after oral administration, chitosan-coated nanoparticles (EANP@CS) were loaded. The average EANP@CS size was between 20 and 62 nm, with a decrease in DNA fragmentation, MDA, and NO in the liver and serum. Meanwhile, rats given EANP@CS had higher liver thiol and GSH levels, as well as higher Gpx, catalase, and GST activity than those with nitrites. In rats that received EANP@CS during or following the cessation of nitrite treatment, liver NOS activity was decreased by 7 and 4.9 times, respectively. The rats given nitrite or treated with EANP@CS had increased liver arginase activity. The tissues of the rats administered nitrites showed inflammatory infiltrations of the liver, kidney, and spleen, which were reduced when the animals were given EANP@CS. Moreover, EANP@CS increased the oral bioavailability and decreased the danger of sodium nitrite. This shows that any undesired hazardous ingredient in food can be detoxified using EANP@CS as a therapeutic objective [109].

Mohasseli et al. (2020) studied the effects of the Fe nanoparticle medication delivery system on physiological variables and lemon balm essential oil production (*Melissa officinalis* L.). The investigation was carried out to examine the impact of Fe-NPs (Fe-NPs; 0, 5, 10, 20, 30, and 20 μM) on *Melissa officinalis*’s tolerance to decreased irrigation by an 80, 60, and 40% field capacity (FC). Based on the results, a drought led to a notable decrease in the chlorophyll content, the ratio of the variable to maximum fluorescence (Fv/Fm), and the relative water content. This occurred when increasing malondialdehyde (MDA), H_2_O_2_, proline content, electrolyte leakage, total antioxidant activity (TAA), total phenolic content (TPC), antioxidant power, and enzyme activities of lemon balm. The *Melissa officinalis* essential oil output rose when the irrigation was reduced from 80 to 60% FC, and it declined at a high water deficit of 40% FC. Subsequently, the essential oil output was boosted, and the physiological characteristics of lemon balm were improved by treating the plants with all concentrations of Fe NPs. By lowering the activities of TAA, TPC, and antioxidant enzymes, the Fe NPs also decreased the oxidative stress. Due to the increase in proline accumulation and a decrease in certain antioxidant activities, the Fe nanoparticle treatments had a compensating effect that mitigated the deleterious effects of reduced watering and boosted the drought tolerance of lemon balm. Both reduced watering practices and Fe NPs had a significant impact on the content and production of essential oils in lemon balm [115].

Cu nanoparticles were developed by Seckin et al. because of their affordability and potential impacts on human health, such as the production of blood cells, oxidation, and reduction. This study specifically sought to evaluate the antioxidant capacity and DNA damage prevention capabilities of Cu nanoparticles made from the *Diplotaenia turcica* plant. Based on observation, a potent antioxidant activity was discovered in the antimicrobial properties of the copper nanoparticles against several different bacteria. These included *Staphylococcus aureus* ATCC 29213, *Pseudomonas aeruginosa* ATCC 27853, *Enterococcus faecalis* ATCC 29212, *Bacillus subtilis* ATCC 6633, *Bacillus cereus* ATCC 10876, *Escherichia coli* ATCC 25952, and *Candida albicans*. Furthermore, the Cu NPs/Dt exhibited a greater antimicrobial impact than the positive control antibiotic against several bacteria. The collected DNA scans revealed that, depending on the concentration, there is a possibility of preventing DNA breakage [112].

Pasha et al. (2020) showed that date seed nanoparticles (DSNPs) were manufactured using an acid (HCl) hydrolysis process, namely, an HCl concentration of 38% for 4 days. The results showed that the particle sizes ranged from 50 to 150 nm. This indicated that the S:L ratio (40:1 mg/mL) and treatment duration were the two most important independent parameters affecting the TPC, TFC, and antioxidant activities in UAE of DSNPs using a water-based solvent (9 min). Meanwhile, the US amplitude/power (90%) and methanol concentration (80%) had the greatest impact on the methanol-based UAE of DSNPs. The relevance and validity of each RSM model were further confirmed using Minitab’s response optimizer, and the produced projected values were similar to the actual results. The three phenolic acids 3,4-dihydroxy benzoic, ferulic, and p-coumaric acids were the most prevalent phenolic chemicals found in the DSNPs. In addition to documenting the ideal UAE conditions for maximizing the extraction of polyphenolic components from DSNPs and boosting their antioxidant properties to be employed in food applications, an effective technique for synthesizing DSNPs was also established [126].

The one-pot synthesis of orange pectin-encapsulated Fe_3_O_4_ nanoparticles (Fe_3_O_4_@Pectin NPs), produced by co-precipitation of Fe(II)/(III) ions in alkaline solution, was carried out by Zhang et al. (2022). Magnetic nanoparticles were created due to the activity within the pectin network. By using the MTT test, the anti-liver cancer characteristics of the Fe_3_O_4_@Pectin NPs significantly eradicated the cancer cell lines of pleomorphic hepatocellular carcinoma (SNU-387), hepatic ductal carcinoma (LMH/2A), Morris hepatoma (McA-RH7777), and Novikoff hepatoma (N1-S1 Fudr). Furthermore, the Fe_3_O_4_@Pectin NPs had an IC50 of 8, 13, 10, and 7 mg/mL against the pleomorphic hepatocellular carcinoma (SNU-387), the hepatic ductal carcinoma (LMH/2A), the Morris hepatoma (McA-RH7777), and the Novikoff hepatoma (N1-S1 Fudr) cancer cell lines. The antioxidant activity of the Fe_3_O_4_@Pectin NPs was assessed using the DPPH technique. The IC50 value of the Fe_3_O_4_@Pectin NPs indicated a strong antioxidant activity. Meanwhile, the recent nanoparticles appeared to have an antioxidant impact that prevents human liver cancer [114].

In 2020, Xiao, Yu et al. conducted a chitosan nanoparticle drug delivery system for genistein. The findings showed that genistein greatly increased its water dispersibility and antioxidant activity in the aqueous phase, and photostability against UV radiation when it was enclosed in zein nanoparticles. A sustained release property of genistein contained in zein nanoparticles was also demonstrated. Furthermore, it was discovered that a zein/carboxymethyl chitosan (CMCS) coating considerably improved the encapsulation effectiveness of genistein, and this effect was more obvious when the complex nanoparticles were cross-linked with calcium ions than when zein was used as the sole encapsulant. Additionally, CMCS coating considerably improved the thermal and storage durability of the produced nanoparticles compared to zein nanoparticles without biopolymer coating, and also postponed the release of genistein. The phenomena mentioned abovewas a proposed mechanism for the production of zein and CMCS nanoparticles for the encapsulation of genistein. The current study’s findings support the notion that encapsulating genistein in zein/CMCS nanoparticles is a potential strategy for enhancing its water dispersibility, antioxidant activity, photostability against UV light, and controlled release for food and medicinal applications [127].

Another study related to genistein nanoparticles was performed by Pool, Héctor, et al. in 2018. The findings demonstrated that positive surface charge (+9.54 mV) was obtained with small, spherical particles (about 33 nm in size). Genistein was effectively encapsulated with a 51% efficiency according to infrared measurements. It was also noted that the technique improved genistein’s aqueous dispersibility and that the cumulative release of genistein was pH-dependent. More significantly, data obtained after encapsulation revealed that Gen potentiated its antioxidant and antiproliferative effects on HT29 human colon cancer cells by controlling endogenous antioxidant enzymes and H_2_O_2_ production, which simultaneously activated two different cell death processes (apoptosis and autophagy), in contrast to free genistein, which only activated one (apoptosis) in a lower proportion. Our data suggest that Gen-PEG-SiHNM may be employed as an alternate therapy for colorectal cancer in the near future [121].

### 5.4. Particle Size Reduction

Putra et al. (2018) investigated how the particle size affected the amount of oil produced and the antioxidant activity during modified supercritical carbon dioxide and Soxhlet extraction. According to the results, the extractions of the modified supercritical carbon dioxide, Soxhlet, and antioxidant activities with values of 15.53%, 36.28%, and 93.43% produced the greatest yield extract at 425 m (62.21%). With an inaccuracy of less than 5%, the single-sphere model completely suited the experimental results of the modified supercritical carbon dioxide. The greatest diffusivity coefficient was 6.794 × 10^12^ m^2^/s under operating parameters of 10 MPa, 40 °C, and a 425 m particle size [120].

Astaxanthin (AXT) was encapsulated in ethyl cellulose by Tirado et al. (2019) using supercritical emulsions extraction (SEE) technology (EC). The use of 1.0% mass of EC in the oily phase and 0.1% mass of surfactant in the water phase produced the best emulsion formulation. Under these circumstances, spherical nanocarriers with an unwrinkled and smooth surface were produced with a size of 242 nm and a poly dispersity index of 0.16, and 90% of the EC mass was recovered. When AXT was encapsulated under the same circumstances, a higher carrier mean size of 363 nm (poly dispersity index of 0.31) was observed, yielding an encapsulation efficiency of 84%. The carriers had a good antioxidant capacity and were loaded with 21 mg/g of AXT, which is equivalent to 3900 M Trolox when measured in Trolox equivalent (Trolox equivalent per kilogram of pure AXT). Based on a 10 h incubation period, 70% of the entire encapsulated AXT was released, according to in vitro release profiles measured in a simulated intestinal fluid (SIF) at pH 7.2 and 310 K [128].

Jiang et al. (2020) examined how the particle size affected a ginseng IDF’s physicochemical characteristics, antioxidant activity, and structural and thermal analyses. Through careful grinding, an IDF powder with a median particle diameter of 15.83 m was created. The brightness, water holding capacity, and solubility were improved with the ginseng IDF reduction. Furthermore, the mass, tapped density, Carr index, and Hausner ratio decreased as the particle sizes shrank. Particle size reduction also increased the mineral and phenolic content’s extractability, thereby improving the DPPH radical scavenging activity and ferric-reducing antioxidant capacity. Although the rate of hemolysis inhibition was elevated, the greater polyphenol extraction with a smaller particle size caused a decrease in the hemolysis percentage of mouse erythrocytes. The particle size also affected the thermal stability of the ginseng IDF powders.

The effects of the particle size and corn oil on the bioavailability of carotenoids and the antioxidant capacity of SUPF powders during in vitro digestion were explored by Lyu et al. (2021). Compared to 18 mesh-sized powder (18 MP), the total carotenoid relative bioaccessibility (TCRB) of 100 mesh-sized powder (100 MP, 15.46%) was greater. The TCRB increased by 108.35% (18 MP) and 88.55% (100 MP), respectively, with the addition of 2% corn oil. The primary carotenoid monomers in SUPF were lutein (27,160 g/100 g) and -carotene (5192 g/100 g), and they were substantially linked with the DPPH radical scavenging activity of the digestive supernatant (*p* = 0.05). Furthermore, maize oil boosted the 18 MP’s DPPH radical scavenging capacity by 96.54% [117].

Azeem et al. (2021) analyzed the nutritional content and the microstructural, physicochemical, and antioxidant characteristics of orange- and purple-fleshed sweet potato flour (SPF) with particle sizes 75–355 μm. They were influenced by the particle size distribution. It was discovered that the protein content, capacity for water absorption, and water solubility index of both SPFs were reduced with the decreasing particle size, while the starch, reducing sugar, and crude fat increased. The purple-fleshed SPF had a greater TPC (8.25–9.27 mg GAE/g DW) than the orange-fleshed SPF (0.22–0.28 mg GAE/g DW). Meanwhile, the orange-fleshed SPF had higher vitamin C contents (23.59–29.26 mg/100 g DW) than the purple-fleshed SPF (4.24–4.89 mg/100 g DW). The greater TPC and vitamin C levels contributed to the steady increase in the antioxidant capabilities of both SPFs as the particle size decreased. Every SPF fraction displayed an uneven surface, and to create unique food items with the appropriate nutrients and functional qualities, an SPF with a particle size of 75 m can be employed [129].

Odgerel et al. (2021) applied the micro wet milling (MWM) process to develop zero waste, whole sea buckthorn (SBT) juice with pulp, and seeds. The optimum MWM operational conditions of feeding rate at 10 mL/min, a rotational speed at 50 rpm, and an adjusted gap between the two millstones by 43.03 kN were achieved along with a higher yield of minimal particle sizes (D50 of 10.4 μm) of the juice. The MWM SBT juice showed better color, smaller particle size, significantly higher antioxidant properties, and total phenolics in comparison to the mixer-milled and commercial SBT juice. Furthermore, HPLC analysis of SBT juice revealed that higher amounts of low molecular weight organic acids and phenolic compounds were present in the MWM SBT juice than commercial type. The microbiological analysis showed that the initial total viable count of MWM SBT juice was within the permissible limit of standard [130].

Moreover, Hussain et al. (2021) investigated cocoa as an espresso coffee substitute because it has approximately 17% less caffeine content. Theobromine is a polyphenol that contributes to the flavor and aroma of cocoa goods and is a source of antioxidants. To create a concentrated cocoa drink that closely resembles espresso coffee, various fat concentrations and grinding levels were examined. Based on the results, better caffeine content, theobromine, antioxidant capacity, and total phenolic content (TPC) of values 3.31 g/mL, 34.26 g/mL, 1726.3 M TE, and 193.57 mg/mL GAE) were found in concentrated cocoa drinks manufactured from cocoa beans with 20% fat that was ground at level 50. Fresh-brewed concentrated cocoa beverages contained five different types of volatile chemicals, including alcohols, aldehydes, ketones, esters, acids, and pyrazines, which give the drinks a distinctive scent. These results have implications for the cocoa industry since the concentrated cocoa drink can serve as a substitute cocoa beverage with better nutritional value [131].

Dziki et al. (2021) investigated the oat husk (OH) particle size distribution, antioxidant activity, and phenolic composition. The sterilized husk was efficiently ground into smaller pieces by micronization, and when the rotor and classifier were run faster, the median particle size (d50) dropped from 63.8 to 16.7 m. The phenolic acids ferulic, caffeic, p-hydroxybenzoic, vanillic, syringic, and sinapic acids were discovered in OH. Furthermore, approximately 95% of the phenolic acids were ferulic acids. As the particle size of the micronized husk dropped, the antioxidant activity of the derived extracts increased. Based on the results, chelating power had the greatest half maximal inhibitory concentration (EC50 index), while radical scavenging activity had the lowest [132].

Liu et al. (2021) also explored potential soybean hull usage in high-value products by measuring the number of proteins, dietary fiber, minerals, phenolics, antioxidant activities, and functional characteristics of the samples in five different size fractions ranging from 20 to 140 mesh. With shrinking sizes, the concentrations of protein, ash, soluble sugar, and the majority of amino acids increased. The amounts of bound phenolic compounds and antioxidant activities, soluble sugars, and amino acids in soybean hulls were first reported. The statistically significant increase in free phenolic contents (1.81 to 3.24 mg/g) and antioxidant activity (1.51 to 1.95 mol/g) were determined with decreasing particle sizes. Free antioxidant activities were significantly lower than bound antioxidant activities for the same fraction; however, free phenolic contents were often greater than bound phenolic contents. This showed that some antioxidant activities were not released during neutral extraction. With decreasing size, the number of water-soluble solids significantly increased from 15.08 to 23.71%. Water-holding capabilities significantly declined from 666.72 to 337.47%, when particle sizes decreased significantly. As the sizes rise, the soybean hulls’ final viscosity peak also increases, while blends’ ultimate peak trends are correlated with the amount of water stored. The correlation tests also revealed the relationship between the distinctive qualities of soybean hulls that were statistically significant (*p* = 0.05). This showed that soybean hulls are a useful source for both industrial and functional food uses [133].

The activity of juvenile whiteleg shrimp (*Penaeus vannamei*) was examined by Ghaffarizadeh et al. (2022) nutritionally over eight weeks (1.55 0.04 g, means standard deviation) to determine the impact of dietary selenium nanoparticles (Se-N). After 56 days of the feeding study, samples from the hepatopancreas (HP), hemolymph, and intestines were taken. Growth performance (*p* = 0.011) and feed conversion ratio (*p* = 0.006) of shrimp given diets enriched with Se-N showed positive quadratic trends. With an increase in Se-N in their diet, shrimp showed both linear and quadratic trends in their body selenium levels. Meanwhile, supplementing the diet with Se-N caused a rise in the plasma levels of phenoloxidase (PO), catalase (CAT), superoxide dismutase (SOD), and glutathione peroxidase (GPx), which all exhibited positive quadratic trends. Malondialdehyde (MDA) levels considerably reduced and followed a quadratic trend in the HP of shrimp given Se-N (*p* = 0.012). In the relative abundance of prophenoloxidase, lysozyme, and penaeidin-3 genes mRNA transcript in the HP of shrimps given diets enriched with Se-N, both linear and quadratic trends were observed [134].

In the study by Park et al., the effectiveness of flour mixtures for creating noodles was examined alongside the antioxidant capabilities of purple wheat branpurple wheat bran. For smaller particles compared to bigger ones, the damaged starch and swelling capacity of the bran was greater. Blends’ SRC values for water and sodium carbonate rose as bran particle size dropped. The cooked noodles’ stiffness and springiness were measured to be greater the smaller the bran particles were. The antioxidant activity of noodles prepared with mixes showed greater incorporation of the tiny bran particles than the big particles into noodle sheets. At greater blend ratios, small bran particles significantly improved the quality and antioxidant activity of the noodles. The capability of flour containing purple-colored wheat bran to make noodles was improved by the decrease in the bran’s particle size. This is due to an increase in the amount of bran used to make noodles with health advantages [135].

## 6. Discussion and Author Perspective

### 6.1. An Overview

Antioxidants are substances that can inhibit, delay, or prevent the oxidation processes in other substances that are brought on by the presence of radicals. Moreover, radicals are substances with an unpaired electron in their structure, making them stoichiometrically unstable. This instability causes the formation of radicals, also known as oxidizing agents, which are capable of oxidizing other substances, including protein complexes in the human body. By delivering electrons to the radical structure mechanically to become more stable and harmless, antioxidants can stop the oxidation of other chemicals that are caused by radicals [136].

There is a need to be concerned about the existence of free radicals that enter the body, causing anomalies or abnormalities in the physiological activities of the human body. When the body’s antioxidants are unable to protect against radicals that enter the body because they outnumber the antioxidants, this becomes even more harmful (i.e., oxidative stress). It has been demonstrated that oxidative stress can hasten the onset of degenerative illnesses that affect humans [137].

Antioxidants are compounds that can be used as preventative measures in the prevention of different illnesses. However, there is a major concern, since physicochemical constraints such as solubility significantly impair their efficacy. Therefore, several investigations have been conducted to solve the poor solubility problem [72].

Generally, two types of approaches, namely, chemical and physical techniques have been developed to promote solubility. Several modifications, such as salt, co-crystallization, cyclodextrin inclusion complex creation, and structural alteration or synthesis of medicinal compounds can all be used to boost chemical solubility. Meanwhile, the different ways to modify the physical properties of an object include solid dispersion, amorphous solid dispersion, co-amorphous, particle size reduction, and drug delivery systems using nanoparticles.

The methods of modifying the physical properties, as stated above, are used to enhance the solubility of antioxidant compounds, according to a previous review.

### 6.2. Molecular Mechanism

#### 6.2.1. Amorphous Solid Dispersion

**Figure 9:**
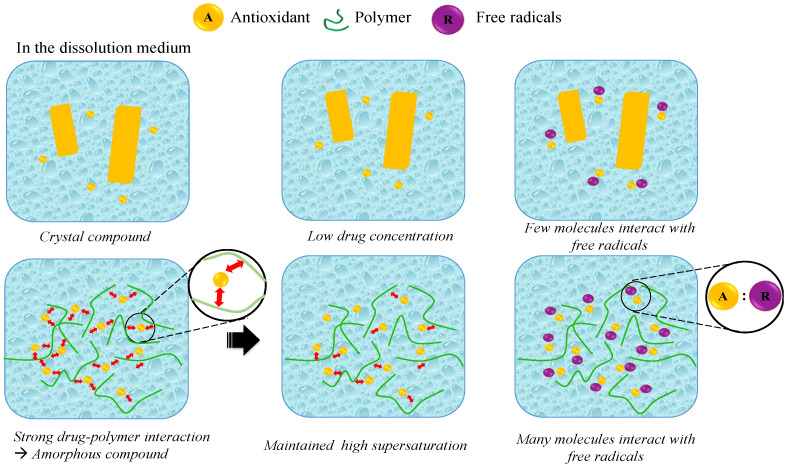
The schematic illustration of the antioxidant activity mechanism of the drug in amorphous solid dispersion.

The effectiveness of this method is demonstrated in Table 2 and Section 5.1, where each study showed a meaningful impact on boosting the antioxidant activity. Since crystallized compounds often have limited water solubility, the solid dispersion approach technique combines crystalline active ingredients with amorphous polymers, with high water solubility. The active ingredient and polymer are mixed physically or chemically in the production process, whether in a molecular or nonmolecular state. This allows interactions to form between their molecules, where potential, stable, and strong interactions occur when hydrogen bonding interactions are formed. Furthermore, the characterization of the active ingredient and polymer complex was shown in the development of solid dispersion. Based on the amorphous pattern of the results of the crystallography and differential scanning calorimetry, the active ingredient was molecularly dispersed in the polymer phase. Therefore, the amount of active ingredient that is in a molecular state and is actively playing the role of a reductant in free radicals increases, which improves the solubility of the active ingredient. This is because when the polymer contacts the solvent medium and slowly starts to dissolve, the active ingredient will also dissolve. This is characterized by a lowering of the IC50 value or an increase in the antioxidant activity [138,139]. Therefore, a mechanism of the antioxidant compound’s dissolution from an amorphous solid dispersion was proposed based on the molecular state characterized, as shown in Figure 9. In the amorphous solid dispersion, the drug–polymer interactions were relatively strong in the solid state. The strong interactions between the drug–polymer can reduce the tendency toward phase separation in the presence of water, and offer polymer-controlled dissolution of the drug. When dispersed into the dissolution medium, the drug is rapidly released due to the monomolecular dispersion. The interaction between the drug and polymer can maintain the high supersaturation of the drug; therefore, the amount of drug interaction with the free radicals in the dissolution medium was very high. The crystalline state of the drug showed a low equilibrium solubility, which reduced the number of drug interactions with the free radicals in the dissolution medium.

#### 6.2.2. Co-Amorphous

**Figure 10:**
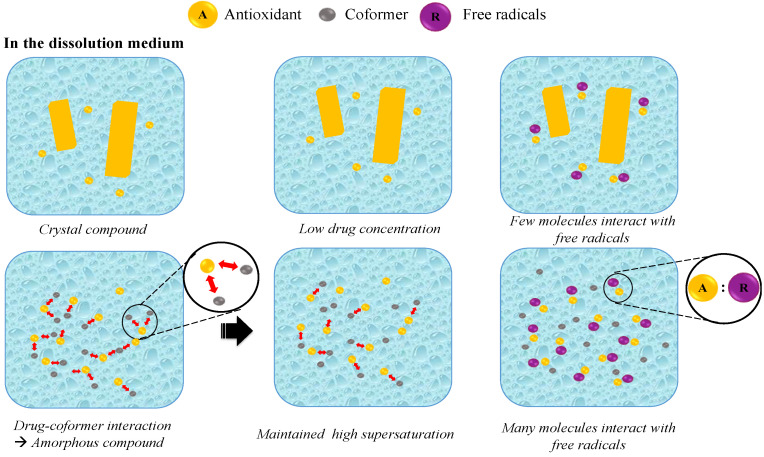
The schematic illustration of the antioxidant activity mechanism of the drug in a co-amorphous system.

The effectiveness of this strategy is demonstrated in Table 2 and Section 5.2, where the majority of the results point to an increase in the activity, including the antioxidant of the compound under development. This kind of modification process for boosting antioxidant activity has the propensity to follow the pattern of the solid dispersion method. However, the focus is on transforming the crystal’s structure to amorphous, with the addition of materials that can alter the active ingredient’s shape in an amorphous state without the use of a dispersion system. This causes instability in this kind of modification, because when the active component and the amorphous conformer are separated from one another, compounds with a strong tendency for recrystallization may perform it. Therefore, this approach is created by two systems, namely, by dispersing the synthesized amorphous form into a dispersion system, also known as an amorphous solid dispersion. Generally, the solubility increase mechanism also occurs because the active ingredients are being transported when the polymer dissolves in the medium. This enables many of the active ingredients to be in a molecular state and actively perform their role as free radical reducing agents [6,60,140,141]. A mechanism of the antioxidant compound dissolution co-amorphous system was proposed based on the molecular state characterized, as shown in Figure 10. It was discovered that the strong interactions between the drug co-former can inhibit the recrystallization of the drug during storage and reduce the tendency toward phase separation in the presence of water. When dispersed into the dissolution medium, the drug is rapidly released due to the fact of its amorphization. The presence of the co-former also suppresses the water contact with the drug, because the hydrophilic part of the drug was already occupied via hydrogen bonding with the co-former. The interaction between the drug and co-former can maintain the high supersaturation of the drug; hence, the amount of the drug that interacted with the free radicals in the dissolution medium was very high. Meanwhile, the crystalline state of the drug showed a low equilibrium solubility, which reduced the number of drug interactions with the free radicals in the dissolution medium.

#### 6.2.3. Nanoparticle Drug Delivery System and Particle Size Reduction

**Figure 11:**
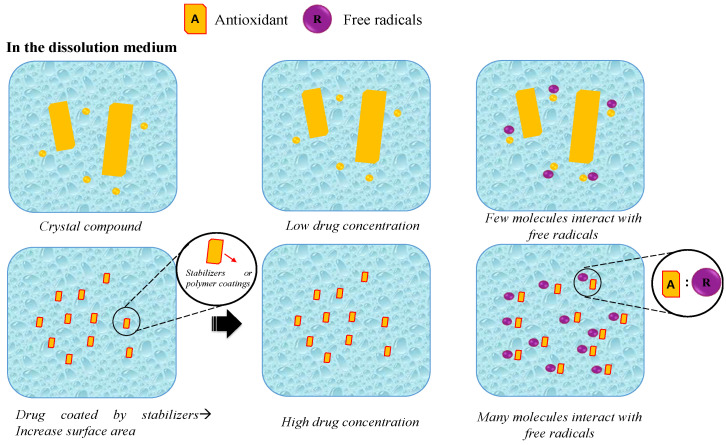
The schematic illustration of the antioxidant activity mechanism of the drug in nanoparticle drug delivery system and particle size reduction.

The effectiveness of this strategy is demonstrated in Table 2 and Section 5.3 and Section 5.4. The primary principle of the nanoparticle drug delivery system is particle size reduction. In this method, the small particle size allows for a large surface area of the particle and a large contact area between the active ingredients, as well as the solvent to increase the wettability of the particles and the active ingredient’s rate of dissolution. This approach leads to an increase in the antioxidant activity of the modified compounds. Secondly, nanoparticles can deliver controlled and targeted release effects, which is very useful for extending the duration of an effect through an extended-release system. Meanwhile, the nanoparticle drug delivery system approach can deliver a targeted delivery system with the addition of targeting ligands to provide a selective effect [142]. A mechanism of antioxidant compound dissolution from a nanoparticle drug delivery system and particle size reduction was proposed based on the molecular state characterized, as shown in Figure 11.

## 7. Conclusions

The results show that the chemical or physical strategies used to increase the solubility and activity of antioxidant compounds are effective. Future advancements can make these antioxidants the primary therapeutic choice or an adjuvant therapy in the diagnosis, treatment, and prevention of a disease, thereby becoming more beneficial to community needs.

## Figures and Tables

**Table 1:** Common Active Pharmaceutical Antioxidant Compounds.

No	Antioxidants	Structures	Antioxidant Activity	Solubility in Water	References
1	Alpha Mangostin	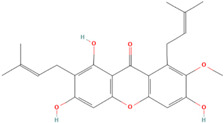	66.63 ± 34.65 µg/mL	2.03 × 10^−4^ mg in 1 L at 25 °C	[13]
2	α-tocopherol	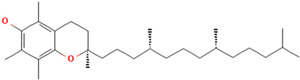	0.059/0.01 mM	Insoluble in water	[14]
3	Ascorbic acid	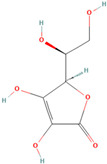	8.9 ± 0.1 µg/mL	Soluble in water	[15]
4	Buthyl Hydroxy Toluene (BHT)	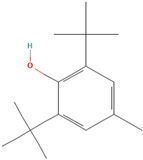	0.020 ± 0.001 µg/mL	Insoluble in water	[16]
5	Buthyl Hydroxy Anisole	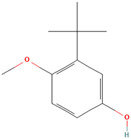	0.035 ± 0.007 µg/mL	Insoluble in water	[16]
6	β-carotene	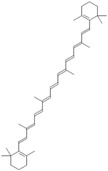	24.99 µg/mL	0.0006 g in 1 L at 25 °C	[17]
7	Curcumin	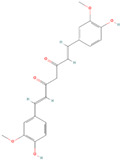	32.86 µM	3.12 mg in 1 L at 25 °C	[18]
8	Catechin	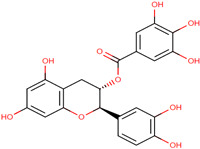	170.3 ± 2.0 µg/mL	0.45 mg in 1 mL at 25 °C	[19]
9	Quercetin	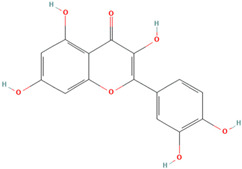	19.3 µg/mL	60 mg in 1 L	[20]
10	Lycopene	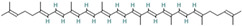	57.93 µg/mL	Insoluble in water	[21]
11	Lutein	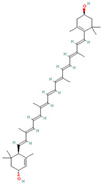	35 µg/mL	Insoluble in water	[22]
12	Tertbutyl hydroquinone (TBHQ)	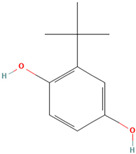	14.9 ± 0.81 mM	Practically insoluble in water	[23]
13	Ferulic Acid	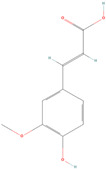	56.4 ± 4.6 µg/mL	0.78 g in 1 L	[24,25]
14	Myricitrin	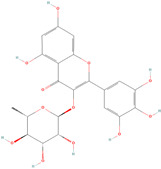	32.7 ± 0.9 µg/mL	Practically insoluble in water	[26]
15	Ethyl Gallate	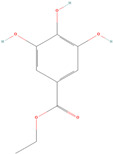	35.3 ± 4.0 µg/mL	Sparingly soluble in aqueous buffers	[26]
16	Resveratrol	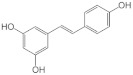	0.49 ± 0.03 mM	3 mg in 100 mL	[27,28]
17	Rutin	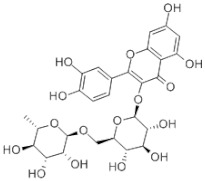	2.77 ± 0.09 mM	12.5 mg in 100 mL	[28]
18	Kaempferol	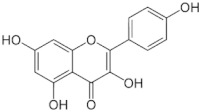	0.82 ± 0.04 mM	0.18 g in 1 L	[28]
19	Myricetin	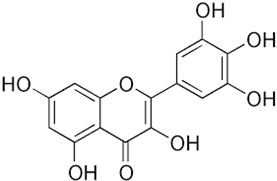	3.66 ± 0.30 mM	Very insoluble (<5 μg in 1 mL) in pure water	[28]
20	Isobavachalcone	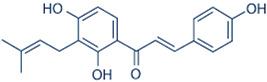	250.8 µg/mL	Insoluble in water	[29]
21	Genistein	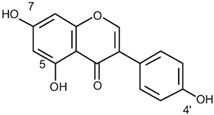	13.6 μM	Insoluble in water	[30,31]

**Table 2:** Water Solubility Improvement Strategies of Poorly Soluble Antioxidants.

No	Technique Modification	Main System: Polymer/ Co-Former/ Co-Amorphous	Active Pharmaceutical Ingredients (Antioxidants)	Methods	IC50 Value Before Modification	IC50 Value After Modification	References
1	Solid Dispersion	silica gel	*Anemarrhena asphodeloides* Bge.	DPPH	-	Improved antioxidant	[98]
2	Solid Disperssion	Silver	Curcumin	DPPH	60%	20%	
3	Solid Dispersion	PE G 6000 and maltodextrin groups	-	CUPric Reducing Antioxidant Capacity (CUPRAC)	13.63 ppm (EC50)	9.00 ppm (EC50)	[99]
4	Solid Dispersion	poly-vinyl-pyrrolidone (PVP) K30	Usnic acid	DPPH	63.867 µg/mL	12.42 µg/mL	[100]
5	Solid Dispersion	Pullulan (PUL)	Rutin	2,2’-azino-bis(3-ethylbenzothiazoline-6-sulfonic acid (ABTS)	13.5 mg/mL	1.75 mg/mL	[101]
6	Solid Dispersion	polyvinylpyrro- lidone K30 (PVP-K30), polyethylene glycol 6000 (PEG 6000) and poloxamer-188 (PLX-188)	ferulic acid	ABTS	58% in 200 µM	50% in 200 µMm	[102]
7	Solid Dispersion	polyethylene glycol 4000 (PEG 4000)	Luteolin (LT)	DPPH	92.28%	66.03%	[10]
8	Co Amorf	nickel chloride–ethanol amine complex	nickel oxide	DPPH	57.42%	41.36%	[103]
9	Co Amorf	Calsium Phosphate	Curcumin	DPPH	49.5%	46.5%	[104]
10	Co Amorf	EHO-85	*Olea europaea* Leaf Extract	ABTS	2220 *±* 102, 1558 *±* 76, and 1969 *±* 114 µmol TE/g	1.62 *±* 0.03, 1.35 *±* 0.06, and 4.07 *±* 0.15 µmol TE/g.	[105]
11	Co Amorf	PVP K-30	Quercetin (3,3′,4′,5,7-pentahydroxil-flavon)	DPPH	1.102 µg/mL	0.714 μg/mL	[72]
12	Co Amorf	nickel chloride [NiCl_2_4H_2_O]	nickel oxide	DPPH	57.42%	32.56%	[103]
13	Co Amorf	SiO_2_	Citrus limon	DPPH and CUPRAC	0.67 ± 0.04 mg/mL	0.66 ± 0.03 mg/mL	[63]
14	Co Amorf	Selenium	imidazolium	ABTS colorimetric assay.	0.55 mg/mL	0.80 mg/mL	[106]
15	Co Amorf	Electrospun Poly(“-caprolactone)	(*Salvia officinalis* L.) Extract	DPPH	55.32%	50.56%	[107]
16	Co Amorf	hydrogen bromide or choline chloride	demythylation of curcumin	DPPH	114 µg/mL	30 µg/mL	[108]
17	Nano Particle	chitosan-coated nanoparticles	sodium nitrites	In-vivo: rat liver and kidneys by scavenging superoxide, hydroxyl anion, and peroxynitrites	-	Improved antioxidant	[109]
18	Nano Particle	ZrO_2_ and TiO_2_ nanoparticles were synthesised from natural resources	zirconia (ZrO_2_) and titania (TiO_2_)	DPPH		76.9%	[110]
19	Nano Particle	gallic acid or octyl gallate	Ochratoxin-A (OTA) is a mycotoxin produced by *Penicillium* and *Asperigillus*	ABTS	44.2 ± 3.0 µg/mg	25.5 ± 2.5 µg/mg	[111]
20	Nano Particle	Cu metal	*Diplotaenia turcica* Plant	DPPH	-	Increased significantly antioxidant activity	[112]
21	Nano Particle	-	leaves extracts of *Phlomis crinita*	DPPH	74.49 ± 1.42	20.51 ± 1.42	[113]
22	Nano Particle	Fe_3_O_4_	orange pectin	DPPH	-	High antioxidant activity	[114]
23	Nano Particle	Fe	Lemon balm (*Melissa officinalis* L.)	DPPH	40%	70%	[115]
24	Particle Size Reduction	PEGylated chitosans	Chtosan	DPPH	-	14.9 mg/mL	[116]
25	Particle Size Reduction	Corn Oil	Powders made from seed-used pumpkin flesh (SUPF)	DPPH	(28.78 µmol TE/L	(3.29 µmol TE/L)	[117]
26	Particle Size Reduction	-	wheat bran	DPPH, FRAP, and (ORAC)	14.58 ± 2.1 µM	3.03 ± 0.34 µM	[118]
27	Particle Size Reduction	-	persimmon seed, peel, and calyx powders	DPPH	65%	35%	[119]
28	Particle Size Reduction	-	peanut skin	DPPH	500 μm = 78.23%	425 μm = 90%	[120]
29	Naoparticle	PEGylated silica	Genistein	Oxygen radical absorbance capacity (ORAC) and the Trolox equivalent antioxidant capacity (TEAC)		Improved significantly antioxidant activity	[121]

## Data Availability

Not applicable.
